# Restoration of Oligodendrocyte Pools in a Mouse Model of Chronic Cerebral Hypoperfusion

**DOI:** 10.1371/journal.pone.0087227

**Published:** 2014-02-03

**Authors:** Jamie McQueen, Michell M. Reimer, Philip R. Holland, Yasmina Manso, Mark McLaughlin, Jill H. Fowler, Karen Horsburgh

**Affiliations:** 1 Centre for Neuroregeneration, University of Edinburgh, Edinburgh, United Kingdom; 2 Centre for Cognitive Ageing and Cognitive Epidemiology, University of Edinburgh, Edinburgh, United Kingdom; 3 School of Veterinary Medicine, Division of Veterinary Biosciences, University of Glasgow, Glasgow, United Kingdom; Massachusetts General Hospital/Harvard Medical School, United States of America

## Abstract

Chronic cerebral hypoperfusion, a sustained modest reduction in cerebral blood flow, is associated with damage to myelinated axons and cognitive decline with ageing. Oligodendrocytes (the myelin producing cells) and their precursor cells (OPCs) may be vulnerable to the effects of hypoperfusion and in some forms of injury OPCs have the potential to respond and repair damage by increased proliferation and differentiation. Using a mouse model of cerebral hypoperfusion we have characterised the acute and long term responses of oligodendrocytes and OPCs to hypoperfusion in the corpus callosum. Following 3 days of hypoperfusion, numbers of OPCs and mature oligodendrocytes were significantly decreased compared to controls. However following 1 month of hypoperfusion, the OPC pool was restored and increased numbers of oligodendrocytes were observed. Assessment of proliferation using PCNA showed no significant differences between groups at either time point but showed reduced numbers of proliferating oligodendroglia at 3 days consistent with the loss of OPCs. Cumulative BrdU labelling experiments revealed higher numbers of proliferating cells in hypoperfused animals compared to controls and showed a proportion of these newly generated cells had differentiated into oligodendrocytes in a subset of animals. Expression of GPR17, a receptor important for the regulation of OPC differentiation following injury, was decreased following short term hypoperfusion. Despite changes to oligodendrocyte numbers there were no changes to the myelin sheath as revealed by ultrastructural assessment and fluoromyelin however axon-glial integrity was disrupted after both 3 days and 1 month hypoperfusion. Taken together, our results demonstrate the initial vulnerability of oligodendroglial pools to modest reductions in blood flow and highlight the regenerative capacity of these cells.

## Introduction

Oligodendrocytes are the myelin producing cells of the CNS and are critical for maintaining and regulating the myelination of axons. Oligodendrocyte survival and the integrity of myelinated axons is essential for maintaining saltatory conduction, neuronal communication and normal cognitive function (for review see [1]). A single oligodendrocyte can myelinate up to 50 axonal segments [2] and thus damage to individual oligodendrocytes could have a major effect on myelination of axons and efficiency of the relay of information.

Oligodendroglia appear to be particularly vulnerable to blood flow reductions and in animal models of cerebral ischemia and severe hypoperfusion a marked loss of oligodendrocytes occurs rapidly in response to severe reductions in blood flow [3]–[6]. Additionally, *in vitro* models of hypoxia and oxygen-glucose deprivation, common pathways in cerebral ischaemia, have demonstrated the susceptibility of oligodendrocytes to these conditions [7], [8] and it is now thought that damage to oligodendrocytes is mediated by oxidative stress, inflammation and excitotoxicity (for review see [9]). Indeed, damage to myelinated axons and oligodendrocytes is prominent in various conditions in which cerebral blood flow is compromised such as the ageing brain [10], [11], Alzheimer's disease [12], [13] and stroke [4], [14] and may contribute to a functional impairment.

Despite the initial degeneration of oligodendrocytes following injury there is now evidence to indicate that oligodendrocyte precursor cells (OPCs) can proliferate and differentiate and as a result may serve to replenish the loss of damaged oligodendrocytes and potentially repair functional deficits. In neonatal models of hypoxic-ischaemic injury, cell proliferation is increased and new oligodendrocytes are generated up to several weeks after the initial injury [15]. In the adult brain there also appears to be an endogenous capacity to generate new oligodendrocytes in response to cerebral ischaemia. In models of focal cerebral ischaemia when either blood flow is restored with reperfusion or in the peri-infarct region where there is sufficient collateral flow, increased numbers of OPCs are detectable [6], [16], [17]. Interestingly, in aged human brain, increased numbers of oligodendrocytes and OPCs occur in areas adjacent to white matter disruption where blood flow is compromised [18] and increased numbers of oligodendrocytes have been demonstrated in cases of vascular cognitive impairment [19]. Together these studies suggest that OPCs may respond to reduced cerebral blood flow in an attempt to ameliorate white matter damage.

There are a number of mechanisms that may regulate OPC proliferation and differentiation. Relevant to blood flow alterations both glutamate and ATP, the extracellular levels of which may be increased with reduced blood flow, have been shown to be important in regulating OPC proliferation and differentiation [20], [21]. In addition, recent studies have identified a role for a G-coupled protein receptor, GPR17, as an important mediator of OPC differentiation and white matter repair [22], [23]. GPR17 has been shown to be expressed by a subset of OPCs [23], [24] and it is thought that these cells may operate as an early sensor of brain damage whereby they are activated by uracil nucleotides and cysteinyl leukotrienes which are increased in response to cerebral ischemia. In support of this, GPR17 positive cells are upregulated in response to cerebral ischemia and associated with oligodendrocyte differentiation [23].

The present study sought to determine whether the pools of oligodendrocytes and OPCs would be influenced by modest reductions in blood flow more akin to those occurring in the ageing brain. We utilised a mouse model of cerebral hypoperfusion induced by permanent bilateral carotid stenosis which we have previously shown to result in diffuse white matter pathology [12], [25], [26]. Importantly, these mice also exhibit impaired spatial working memory [12], [27], providing a link between white matter disruption and cognitive decline. More recently, microarray analysis in mice subject to cerebral hypoperfusion has revealed increased expression of several genes involved in cell proliferation [25] which may underlie a potential white matter repair mechanism. We therefore also investigated the extent of OPC proliferation and differentiation and whether this was mediated by GPR17. In addition, ultrastructural analysis and myelin labelling were carried out to determine whether alterations to oligodendrocyte pools influenced myelin sheath thickness.

## Materials and Methods

### Ethics statement

All procedures were authorised under the Home Office approved Project Licenses, ‘Pathophysiology of Alzheimer's disease: link to cerebrovascular disease’ (licence number 60/3722) and ‘Pathophysiology of vascular cognitive impairment and Alzheimer's disease’ (licence number 60/4350) held by Prof. K. Horsburgh. The licences were approved by the University of Edinburgh's Ethical Review Committee and the Home Office, and adhered to regulations specified in the Animals (Scientific Procedures) Act (1986).

### Animals and surgery

Adult male C57Bl/6J mice (aged 3–5 months old, 25–30 g) were obtained from Charles River Laboratories Inc, UK. Animals were subject to chronic cerebral hypoperfusion as previously described [25]–[28]. In brief, wire microcoils (0.18 mm internal diameter, Sawane Spring Co., Japan) were applied to both common carotid arteries under isoflurane anaesthesia (induced at 5%, and maintained at 1.2–1.6%). A 30 minute interval was left between left and right coil application. Sham-operated animals underwent identical procedures with the exception that coils were not placed around the arteries. Housing of animals and all procedures were carried out in pathogen-free animal units.

### BrdU labelling

Animals from the 1 month cohort were given intraperitoneal injections (35 mg/kg body weight) of 5′-bromo-2′-deoxyuridine (BrdU; Fluka, UK) twice daily for the first 3 days following surgery to label proliferating cells during this period by an individual blinded to surgical group.

### Laser speckle contrast imaging

An additional cohort of animals underwent measurement of cerebral blood flow using laser speckle flowmetry. Animals were anaesthetised with 5% isoflurane in oxygen for 1.5 minutes in an anaesthetic chamber. Animals were then transferred to a stereotaxic frame and their heads were fixed into position. Anaesthesia was maintained at 2–2.5% isoflurane via a nose cone and body temperature was monitored and regulated. An incision was made to expose the skull and the skin overlying the skull was reflected. The skull was moistened using saline and a small amount of water-based gel (37°C) was spread evenly onto the skull. A moorFLPI2 Speckle Contrast Imager (Moor Instruments, UK) was positioned 20 cm above the head. Image sequences were acquired at a resolution of 752×580 pixels and a frequency of 1 frames/second (20 ms/frame). Following stabilisation of perfusion readings, a 2 minute perfusion recording was carried out.

Raw speckle contrast sequences were analysed using moorFLPI2 Review software (v4.0). Regions of interest were consistent between each mouse and made 1 mm to −2 mm from Bregma with care taken to avoid any artefacts on the skull surface. Data were measured in blood perfusion units (PU) and calculated for each mouse as the percentage change relative to baseline (Figure S1).

### Tissue preparation and immunohistochemistry

At 3 days or 28 days post-surgery, mice were deeply anaesthetised with 5% isoflurane and transcardially perfused with 20 ml 0.9% heparinised saline followed by 20 ml 4% paraformaldehyde (PFA) in 0.1% phosphate buffer (PB, pH 7.4). Following perfusion, the brains were removed and post-fixed in 4% PFA overnight. Brains were then transferred to PB and stored overnight at 4°C. The brains were cut along the midline and free-floating 50 µm sagittal sections were cut using a vibrating blade microtome (Hydrax V50, Zeiss, Germany). Sections were stored in cryoprotective medium (30% glycerol/30% ethylene glycol in PB) at −20°C until required. Different cohorts of animals were used due to the sensitivity of some antibodies to tissue fixation (n = 13 sham, 12 hypoperfused and n = 9 sham, 10 hypoperfused for 3 day studies; n = 10 sham, 11 hypoperfused and n = 9 sham, 9 hypoperfused for 1 month studies). Occasionally animals were excluded from analysis if there was an absence of cellular staining or the quality was deemed too poor to perform accurate analysis.

The following primary antibodies were used in this study: anti-BrdU (1∶200, AB6326, Abcam, UK), anti-CC1 (APC 1∶20, OP80, Calbiochem, USA), anti-GFAP (1∶1000, Z0334, Dako UK), anti-GPR17 (1∶200, 10136, Cayman Chemical, USA) anti-Iba1 (1∶100, ab5076, Abcam UK), anti-MAG (1∶100, sc-9543, Santa Cruz, USA) anti-NG2 (1∶100, AB5320, Millipore, UK), anti-Olig2 (1∶500, Ab9610, and 1∶100, MABN50, both Millipore, UK), anti-PCNA (1∶500 ab29, Abcam UK), anti-PDGFRα (1∶100, 558774, BD Pharmingen UK), and anti-PDGFRβ (1∶100, AF1042, R and D Systems, UK). Cy2, Cy3, DyLight 488 and Alexa Fluor 488 and 647 (all 1∶200) conjugated secondary antibodies were purchased from Jackson ImmunoResearch Laboratories Inc (USA). Alexa Fluor 488 and 546 conjugated secondary antibodies (1∶500) were purchased from Life Technologies Ltd (UK). Double labelling experiments were carried out to confirm the cellular specificity of antibodies used for OPC and oligodendrocyte labelling. Non-specific labelling was blocked using 3% normal serum and sections were incubated in primary and secondary antibodies overnight at 4°C. Sections were mounted onto SuperFrost slides and mounted using Vectashield hard set mounting medium containing the nuclear stain 4′,6-diamidino-2-phenylindole (DAPI) (H-1500, Vector Laboratories, USA).

For BrdU labelling experiments, an additional antigen retrieval step was required. Following 3 washes in PBS, sections were incubated for 30 minutes in 2M HCl at 37°C to allow denaturation of DNA. Following this, sections were given three 5 minute washes in 0.1 M sodium borate buffer (Na_2_B_4_O_7_, pH 8.5). For PCNA labelling, sections were retrieved in 10 mM citrate buffer for 30 minutes at 85°C and blocked using 10% normal serum and 0.5% bovine serum albumin.

Fluoromyelin staining was used to assess myelin integrity following hypoperfusion. Free-floating sections were washed in PBS and mounted onto slides. Following rehydration in PBS, sections were incubated in Fluoromyelin Green (1∶200, Invitrogen) for 1 hour at room temperature. At the outset the conditions were optimised as recommended by the manufacturer. This protocol was determined to be optimal for studying myelin alterations in thick vibratome sections and is a slight modification of that suggested by the manufacturer for thin paraffin sections.

### Confocal laser scanning microscopy and image analysis

Immunolabelled 50 µm sections were imaged using confocal laser scanning microscopy (Zeiss Axioskop LSM 510 or Zeiss LSM710, Zeiss, Germany). All images were acquired using a 20× objective (numerical aperture 0.75) representing an area of 460×460 µm. Images were obtained at a resolution of 1024×1024 pixels. Z-stacks of a minimum of 7 µm were acquired with a step size of 1 µm. The region of the corpus callosum, in sagittal sections, was imaged above the lateral ventricle at the stereotactic co-ordinates, lateral 2.40±0.1 mm, Bregma −1.5±0.1 mm.

Stereological cell counting was performed using ImageJ software (version 1.42q) (National Institutes of Health, USA). Cells were identified based on expression of the immunolabel(s) of interest co-localised with the nuclear stain (DAPI). Cells were manually identified and counted using the ImageJ Cell Counter plugin. To prevent over-counting, cells crossing the left and top sides of the region of interest were included, but any cells crossing the right or bottom boundaries were not counted. Images from the top of the Z-stack were excluded from analysis whilst counts from the bottom image from the stack were included. Cell counts are expressed as the percentage of sham controls or as the number of cells/0.01 mm^3^.

To assess the intensity of GPR17 and fluoromyelin staining, sections were imaged using identical gain and offset settings on the confocal microscope which ensured a common threshold was set in the acquisition of all images. For GPR17 staining, individual GPR17^+^ cells within the corpus callosum were manually outlined and the mean gray value measured and expressed as the average per animal. To assess the intensity of fluoromyelin staining, the corpus callosum was manually outlined and mean gray value measured. All intensity measurements were carried out in triplicate and values averaged. For analysis of MAG immunostaining, images were acquired using identical confocal settings and background subtraction was applied before calculating the percentage area of positive staining. All experiments and subsequent analysis were carried out blind to surgical condition.

### Western blot analysis

In a separate cohort of mice after one month of hypoperfusion (n = 8) or a sham (n = 5) procedure, after decapitation, the brain was rapidly removed, the cerebellum discarded and the remaining brain frozen in liquid nitrogen. Myelin-enriched fractions were prepared by sucrose density centrifugation [29] and the total protein concentration was determined using Pierce BCA Protein Assay Kit (Thermo Fisher Scientific, UK). Proteins were separated by Bis-Tris 4–12% SDS-PAGE (NuPage® Novex®, Life Technologies) and transferred onto PVDF membrane (Immobilon-FL, Millipore). Immunobloting was performed using the Odyssey Infrared Imaging System (LiCor Biosciences, Lincoln, NE, USA). Membranes were blocked 1 hour at room temperature in Odyssey blocking buffer (diluted 1∶1 with phosphate-buffered saline), washed in phosphate-buffered saline–Tween (phosphate-buffered saline with 0.1% Tween) and incubated over-night at 4°C with MBP (1∶10.000, Millipore). After gentle washing, membranes were then incubated 1 hour at room temperature with GAPDH (1∶14.000, Sigma) which was used as a loading control. Membranes were then incubated for 45 minutes with the appropriate fluorescent secondaries (1∶3000, LiCor Biosciences). The western blots were analysed using the LiCOR Bioscience Odyssey system and software. The four MBP isoforms were analysed together and normalized to GAPDH.

### Electron microscopy

To determine whether 3 days or 1 month of chronic cerebral hypoperfusion led to alterations in myelin sheath thickness in the corpus callosum, transmission electron microscopy was carried out.

Animals were transcardially perfused with 5% glutaraldehyde/4% paraformaldehyde, 3 days (n = 5 sham, 6 hypoperfused) or 28 days (n = 7 sham, 7 hypoperfused) after the onset of cerebral hypoperfusion. The brains were then cut into 1 mm thick sections and the corpus callosum manually dissected out. Corpus callosum samples were fixed in 3% glutaraldehyde in 0.1M sodium cacodylate buffer (pH 7.3) for 2 hours and then washed 3 times in the same buffer. Samples were then post-fixed in 1% osmium tetroxide in 0.1M sodium cacodylate. Following dehydration, sections were embedded in araldite resin. Samples were then cut from the midline into the corpus callosum and 60 nm ultrathin transverse sections were cut using a Reichert OMU4 ultramicrotome (Leica Microsystems (UK) Ltd, Milton Keynes) and stained in uranyl acetate and lead citrate. Sections were viewed using a Philips CM120 transmission electron microscope (FEI UK Ltd., Cambridge, England) and images taken at a magnification of 3500× using a Gatan CCD camera (Gatan UK, Oxon, England). To quantify changes in myelin sheath thickness, a lined grid of 0.95×0.95 µm^2^ was overlaid onto each image using Image J software (v1.42q) and fibres were selected for analysis if their myelin sheath was intersected by a grid line. Using the freehand tool, the perimeters of each fibre and axon were manually traced and whole fibre area and axonal area were measured. These values were used to calculate corresponding fibre and axonal diameters. To calculate g-ratio, axonal diameter was divided by whole fibre diameter. For each animal a minimum of 137 fibres were analysed with the observer blind to surgical condition.

### Statistical analysis

Data were analysed using Student's unpaired t-test or the Mann-Whitney U-test depending on parametric or non-parametric distribution. Statistical analysis was performed using GraphPad Prism 5 software (GraphPad Software, San Diego, USA). A probability (p) value ≤0.05 was considered to be statistically significant.

## Results

### Decreased numbers of OPCs and mature oligodendrocytes during the early response to cerebral hypoperfusion

**Figure 1:**
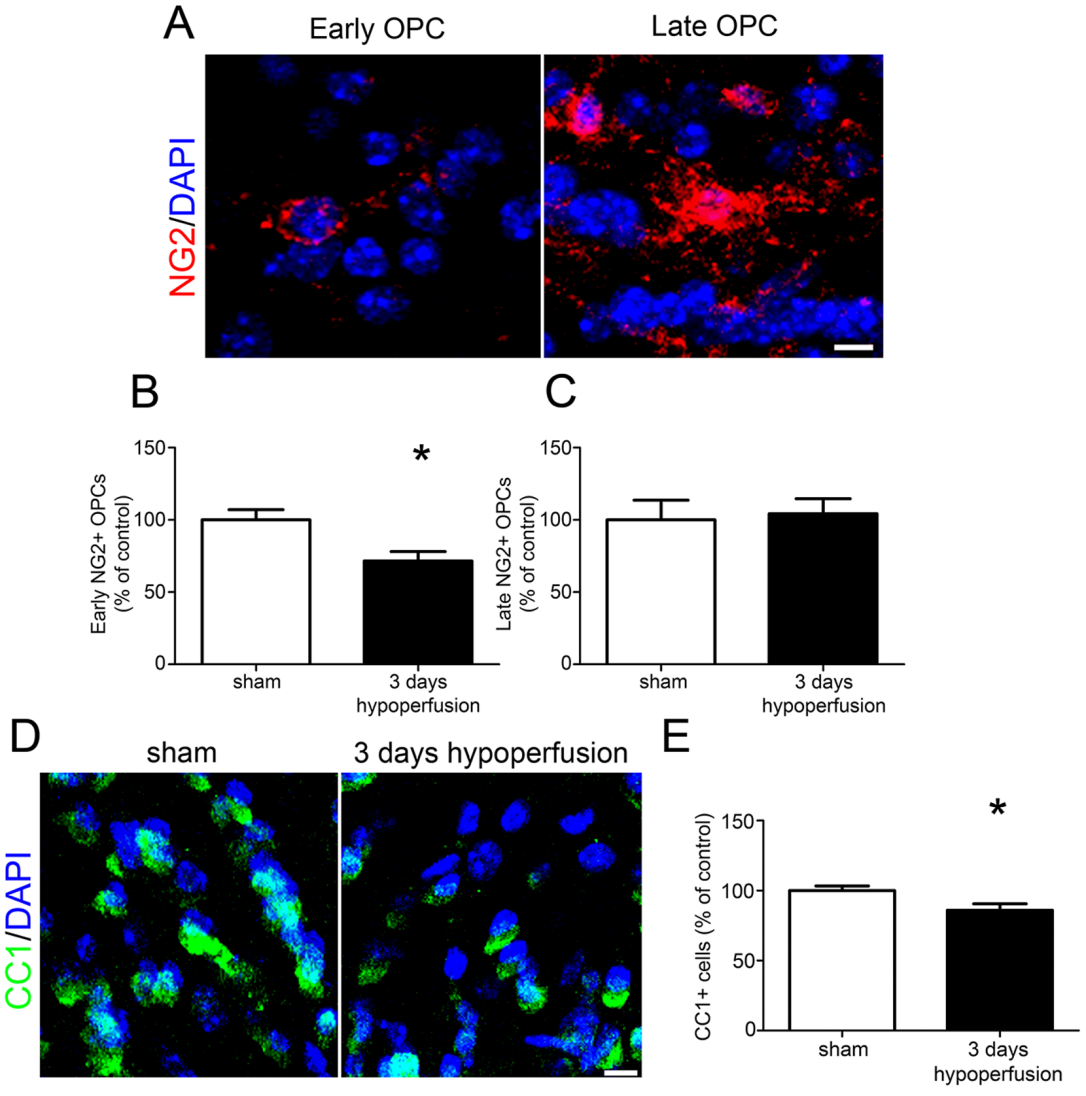
Reduced numbers of OPCs and mature oligodendrocytes after 3 days of chronic cerebral hypoperfusion. (A) Representative confocal images showing morphology of early and late NG2^+^ OPCs in the corpus callosum. (B) A significant decrease in numbers of early NG2^+^ OPCs was found in the corpus callosum after 3 days of chronic cerebral hypoperfusion. (C) No significant differences in numbers of late NG2^+^ cells were observed after 3 days. (D) Representative confocal images of CC1^+^ labelling of oligodendrocyte cell bodies in the corpus callosum. (E) A significant decrease in CC1^+^ oligodendrocytes was found after 3 days of hypoperfusion. n = 13 sham, 12 hypoperfused for NG2 labelling experiments; n = 9 sham, 10 hypoperfused for CC1 labelling. Scale bars = 10 µm * p<0.05.

To assess the acute response of OPCs to chronic cerebral hypoperfusion, NG2 labelling was carried out and numbers of NG2^+^ OPCs were counted. We determined that NG2 specifically labelled OPCs in the corpus callosum through colocalisation with the OPC marker PDGFRα (Figure S2A). In addition, NG2^+^ cells lacked PDGFRβ expression, a marker of pericytes (Figure S2B). Two distinct populations of NG2^+^ cells were identified: one population showed circular immunoreactivity around the nucleus and few processes, corresponding to ‘early’ stage OPCs, whilst the other were more intensely stained and displayed more extensive cellular processes, corresponding to ‘late’ stage OPCs (Figure 1A). Cell counting of both NG2^+^ populations revealed that during the acute response to hypoperfusion, numbers of early NG2^+^ cells were significantly decreased compared to sham controls (p = 0.015) (Figures 1B, S3A). Numbers of NG2^+^ cells displaying late OPC morphology were unchanged after 3 days (p = 0.532) (Figure 1C).

The early effects of cerebral hypoperfusion on mature oligodendrocyte populations were then examined using CC1 immunolabelling (Figure 1D, Figure S3B). This revealed a significant decrease in oligodendrocyte number in the hypoperfused group compared to sham controls (p = 0.027) (Figure 1E). It has been reported that astrocytes may express the CC1 antigen [30], but in this study CC1^+^/GFAP^+^ double labelling determined that only 0.8% of CC1^+^ cells were GFAP^+^ in the corpus callosum (Figure S4A). Furthermore the numbers of astrocytes were unchanged after 1 month of cerebral hypoperfusion (Figures S4B-C). Together these results show that rapid alterations in OPC and oligodendrocyte populations occur in response to modest reductions in cerebral blood flow.

### Restoration of the precursor pool and increased numbers of oligodendrocytes after long term cerebral hypoperfusion

**Figure 2:**
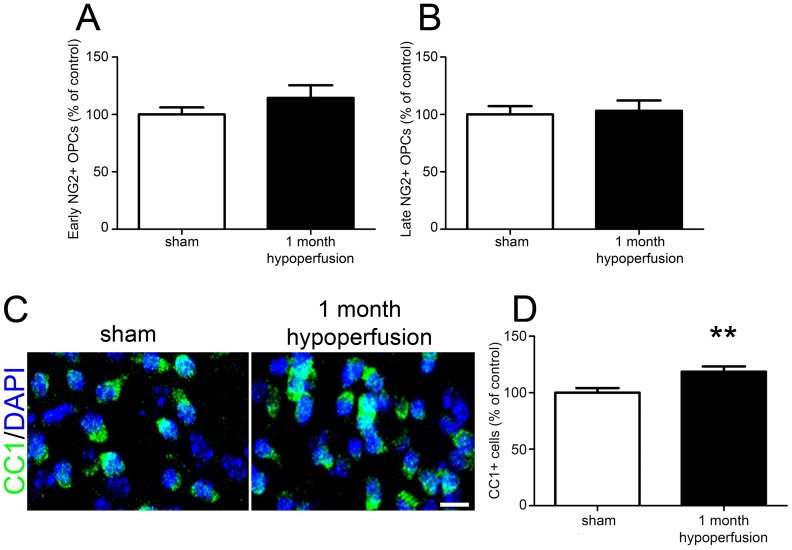
Restoration of the NG2^+^ precursor pool and increased numbers of mature oligodendrocytes after 1 month of chronic cerebral hypoperfusion. (A) No significant differences in numbers of early or (B) late NG2^+^ cells were observed after 1 month of cerebral hypoperfusion. (C) Representative confocal images of CC1^+^ labelling of oligodendrocyte cell bodies in the corpus callosum. Scale bar = 10 µm. (D) A significant increase in CC1^+^ oligodendrocytes in the corpus callosum was observed following 1 month of chronic cerebral hypoperfusion. n  =  10 sham, 11 hypoperfused for NG2 and CC1 labelling. ** p<0.01.

The longer term responses of the oligodendrocyte pools to cerebral hypoperfusion were next investigated. NG2^+^ labelled cells were counted and showed no differences in numbers of early (p = 0.245) or late (p = 0.860) NG2^+^ cells between sham and hypoperfused groups (Figure 2A and 2B respectively). In contrast to the loss of mature oligodendrocytes observed after 3 days of hypoperfusion, CC1^+^ labelling (Figures 2C, S3B) revealed a significant increase (19%) in the number of mature oligodendrocytes in the hypoperfused animals compared to sham operated animals (p = 0.007) (Figure 2D). Together these results indicate that in response to longer term cerebral hypoperfusion a replacement mechanism is acting to restore OPCs and in the case of mature oligodendrocytes, increase numbers of cells.

### Proliferation and differentiation of OPCs in response to cerebral hypoperfusion

**Figure 3:**
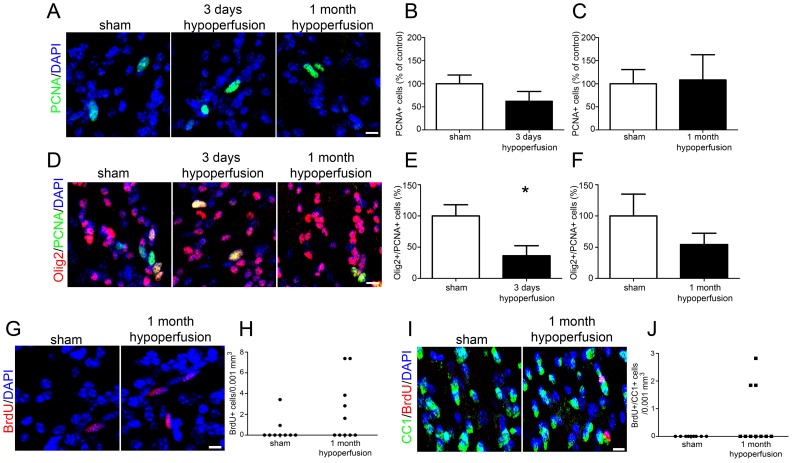
Low numbers of proliferating cells are observed in response to cerebral hypoperfusion however a proportion of newly generated cells differentiate into mature oligodendrocytes within 1 month of cerebral hypoperfusion. (A) Representative confocal images showing PCNA labelling of proliferating cells in the corpus callosum. (B) No significant differences in numbers of PCNA^+^ cells were observed after 3 days or (C) 1 month of cerebral hypoperfusion. (D) Confocal images showing Olig2^+^/PCNA^+^ labelling of proliferating oligodendroglia in the corpus callosum. (E) Decreased numbers of Olig2^+^/PCNA^+^ cells were observed after 3 days of cerebral hypoperfusion. (F) No significant differences in numbers of Olig2^+^/PCNA^+^ cells were found after 1 month of hypoperfusion. (G) Representative confocal images showing BrdU labelled cells in the corpus callosum. (H) BrdU^+^ cells were present in 2 out of 9 (22%) sham control animals but were observed in 5 out of 10 (50%) hypoperfused animals. (I) Representative confocal images showing BrdU^+^/CC1^+^ cells in the corpus callosum. (J) CC1^+^/BrdU^+^ double labelled cells were present in 3 out of 10 (30%) of the hypoperfused cohort but were completely absent from the sham control group. n = 13 sham, 11 hypoperfused for PCNA and Olig2 labelling at 3 days. One animal was excluded from analysis on the basis of poor cellular staining; n = 9 sham, 9 hypoperfused for PCNA/Olig2 and CC1/BrdU labelling at 1 month. Scale bars = 10 µm. * p<0.05.

To characterise levels of proliferation in response to hypoperfusion, PCNA labelling was carried out to determine numbers of proliferating cells at 3 days and 1 month of hypoperfusion (Figure 3A). This showed that overall numbers of proliferating cells were not different between groups at either 3 days (p = 0.20) (Figure 3B) or 1 month (p = 0.564) (Figure 3C). We next sought to determine the extent of OPC proliferation and whether this contributed to the restoration of oligodendroglial pools following hypoperfusion. For technical reasons PCNA/NG2 double labelling could not be carried out therefore PCNA/Olig2 labelling experiments were carried out in 3 day and 1 month cohorts (Figure 3D). Whilst Olig2 is expressed throughout the oligodendrocyte lineage, only OPCs can proliferate and thus express PCNA. The number of PCNA^+^/Olig2^+^ cells were significantly decreased in 3 day hypoperfused animals compared to sham controls (p = 0.02) (Figure 3E) while no differences in proliferating OPCs were observed after 1 month of hypoperfusion (p = 0.323) (Figure 3F). Together these results show that whilst overall levels of proliferation were unchanged after 3 days of hypoperfusion, numbers of proliferating OPCs were decreased in hypoperfused animals compared to controls.

To further investigate the early proliferative responses to cerebral hypoperfusion, animals from the 1 month cohort received injections of BrdU for the first three days following surgery to label all proliferating cells within this period (Figure 3G). BrdU^+^ cells were present in 2 of the 9 (22%) of the sham control group and in 5 out of 10 (50%) of the hypoperfused group (Figure 3H) although the difference between groups was not statistically significant (p = 0.157). BrdU and NG2 double labelling failed to demonstrate any proliferating OPCs (data not shown).

We next sought to determine whether BrdU^+^ cells generated early in response to hypoperfusion had differentiated into mature oligodendrocytes and carried out BrdU^+^/CC1^+^ labelling (Figure 3I). This showed that BrdU^+^/CC1^+^ cells were present in 3 of the 10 (30%) 1 month hypoperfused mice but were completely absent in sham-operated controls (Figure 3J). Although the difference was not statistically significant (p = 0.095), the presence of these double labelled cells in a proportion of hypoperfused animals indicates that OPC differentiation has occurred in a subset of animals in response to reduced cerebral blood flow. CBF responses in individual mice may vary which may account for differences in the proliferative/differentiation responses. An indication of the individual CBF responses was investigated in different cohorts of mice to those used to assess pathology at 3 days and 1 month hypoperfusion (see Figure S1).

### Decreased expression of GPR17 in response to cerebral hypoperfusion

**Figure 4:**
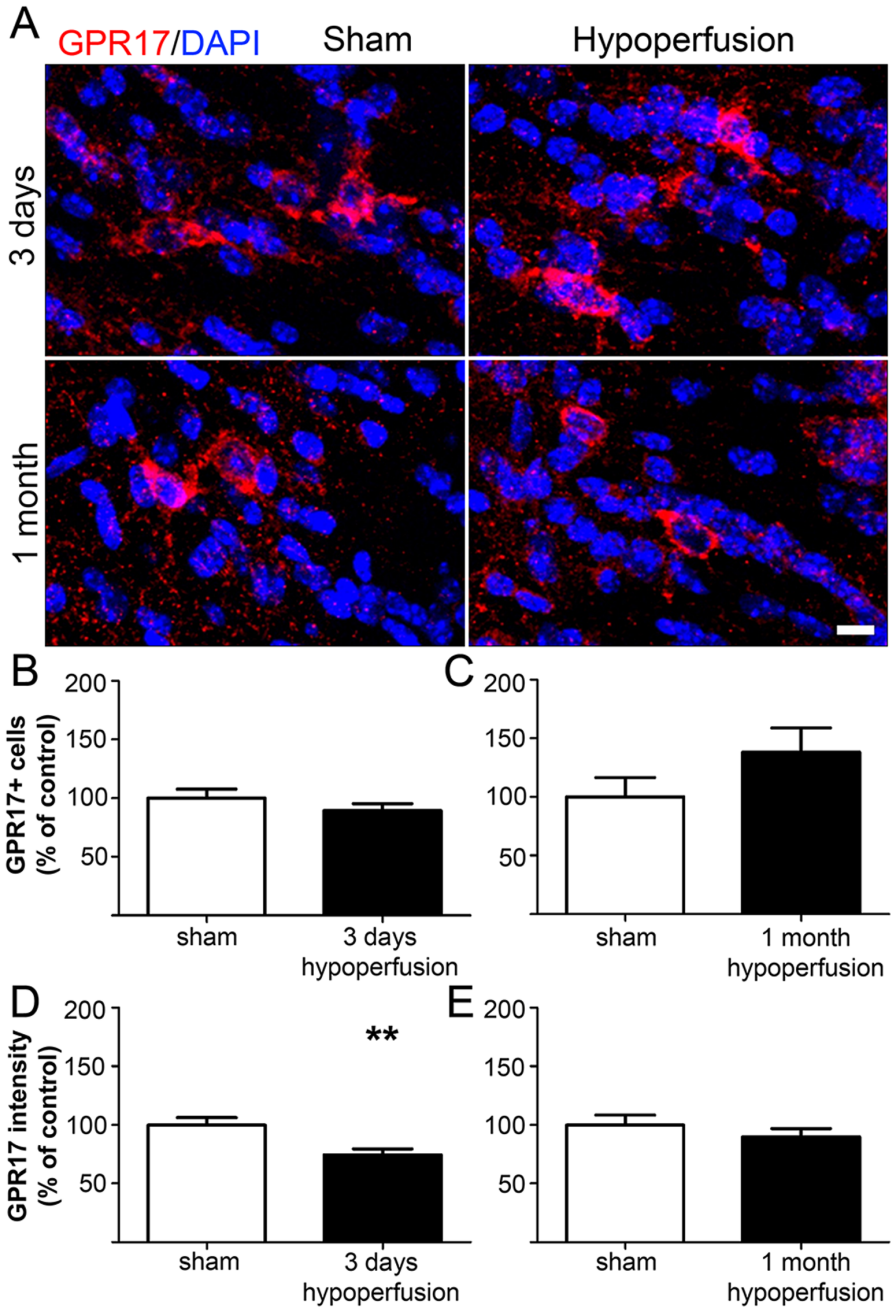
Chronic cerebral hypoperfusion does not alter numbers of GPR17-expressing cells. (A) Representative confocal images showing GPR17 labelling in the corpus callosum following 3 days and 1 month of hypoperfusion. Scale bar = 10 µm. (B) Numbers of GPR17^+^ cells in the corpus callosum were unchanged after 3 days and (C) 1 month of cerebral hypoperfusion. (D) Intensity of GPR17 labelling was significantly decreased after 3 days of cerebral hypoperfusion. (E) No difference in GPR17 labelling intensity was observed after 1 month of cerebral hypoperfusion. n = 13 sham and 11 hypoperfused for 3 day analysis; n = 9 sham and 9 hypoperfused for 1 month analysis. ** p<0.01.

To elucidate a possible mechanism involved in OPC differentiation in response to cerebral hypoperfusion, we analysed the expression of the G protein-coupled receptor GPR17 (Figures 4A, S3D). Previous studies have shown that in the intact brain GPR17 is expressed by premyelinating oligodendrocytes and a subset of OPCs, and receptor activation has been demonstrated to play a permissive role in OPC differentiation [22], [23]. GPR17/NG2 double labelling could not be performed due to the antibodies being raised in the same species therefore we confirmed GPR17 expression by oligodendrocyte lineage cells using Olig2. This showed approximately 64% of GPR17^+^ cells co-expressed Olig2 (Figure S5), which is in agreement with a previous report [23]. Numbers of GPR17^+^ cells were not significantly different between groups at either time point examined (Figures 4B and 4C). We next assessed expression of the receptor, as indicated by the intensity of staining. This revealed a decrease in the GPR17 labelling intensity after 3 days in hypoperfused as compared to shams (p = 0.007) (Figure 4D). However, after 1 month of hypoperfusion there was no difference between groups (p = 0.363) (Figure 4E).

### Impairment of axon-glial integrity, increased microglia and absence of gross myelin alterations in response to cerebral hypoperfusion

**Figure 5:**
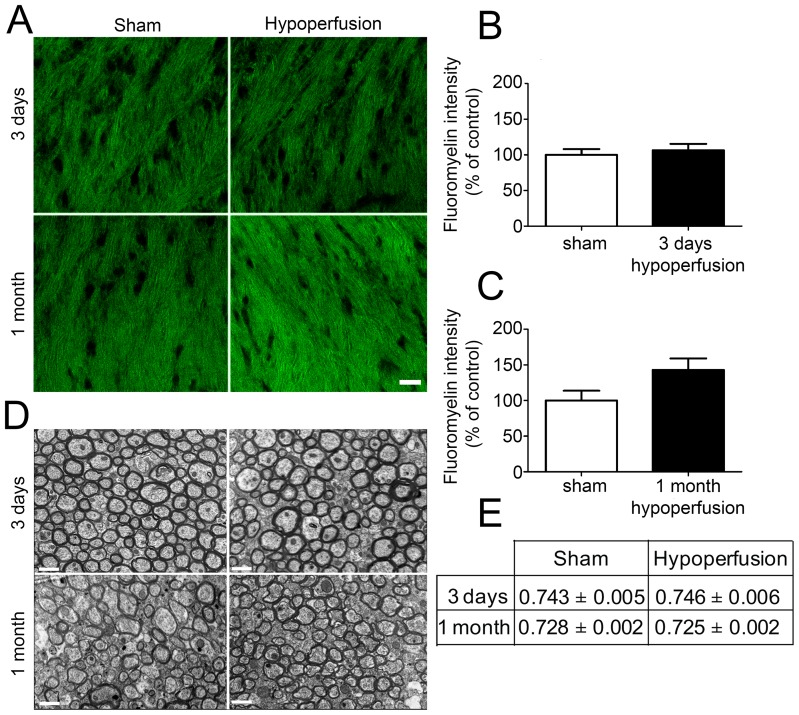
Alterations to numbers of oligodendrocytes as a result of chronic cerebral hypoperfusion does not impact on myelination of axons within the corpus callosum. (A) Representative confocal images showing fluoromyelin labelling in the corpus callosum after 3 days and 1 month of cerebral hypoperfusion. Scale bar = 20 µm (B) No significant difference in fluoromyelin intensity was observed after 3 days of chronic cerebral hypoperfusion. (C) Fluoromyelin intensity was not significantly different between groups after 1 month of cerebral hypoperfusion. n = 13 sham, 12 hypoperfused for 3 day analysis and n = 9 sham, 10 hypoperfused for 1 month analysis. (D) Representative electron micrographs showing myelinated fibres in the corpus callosum after 3 days and 1 month of cerebral hypoperfusion. Scale bars = 1 µm. (E) G-ratio values were unchanged following 3 days and 1 month of hypoperfusion compared to respective sham controls. n = 5 sham and 6 hypoperfused for 3 day analysis; n = 7 sham and 7 hypoperfused for 1 month analysis.

To determine whether alterations in oligodendrocyte numbers may be paralleled by alterations in myelin density following cerebral hypoperfusion, myelin status was assessed using the fluorescent lipophilic dye fluoromyelin (Figure 5A). However, there were no significant differences in staining intensity following either 3 days of cerebral hypoperfusion (p = 0.598) or after 1 month of cerebral hypoperfusion (p = 0.063) (Figure 5B & C). Investigation of myelin-enriched extracts using Western blot analysis additionally indicated that there was no significant difference in MBP levels between sham and one month hypoperfused mice (Figure S6).

To further investigate the myelin integrity, electron microscopy was carried out and measurements of myelin sheath thickness relative to fibre diameter, i.e. g-ratio, were conducted in separate cohorts of 3 day and 1 month hypoperfused animals (Figure 5D). This revealed no significant difference in g-ratio values following both short and long term hypoperfusion compared to respective sham-operated controls (Figure 5E). As a note since the 3 days and 1 month cohorts underwent surgery and tissue processing at different times no statistical comparisons can be made between sham cohorts or the hypoperfused cohorts.

**Figure 6:**
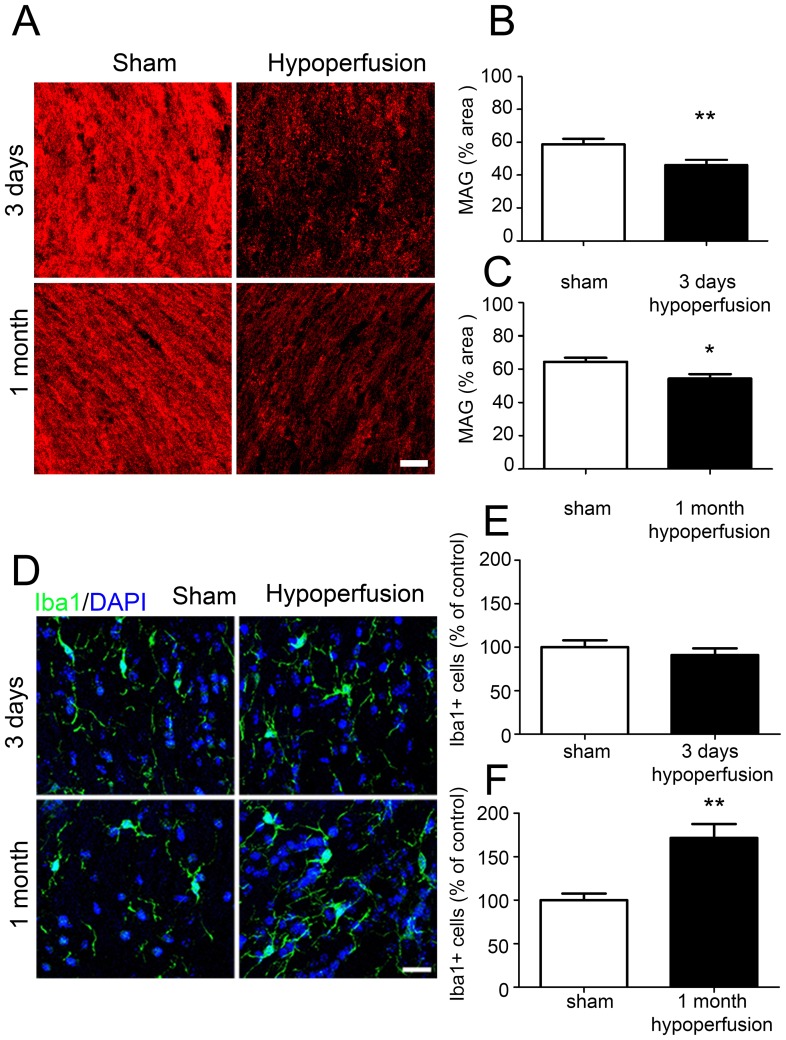
Axon-glial integrity is disrupted and increased numbers of microglia after cerebral hypoperfusion. (A) Representative confocal images showing MAG labelling in the corpus callosum following 3 days and 1 month of chronic cerebral hypoperfusion. Scale bar = 20 µm. (B) A significant decrease in the density of MAG labelling was observed following 3 days of cerebral hypoperfusion compared to sham controls. (C) Similarly, decreased density of MAG was also observed following 1 month of hypoperfusion. n = 12 sham, 12 hypoperfused for 3 day analysis. One animal was excluded due to a lack of positive MAG labelling; n = 9 sham, 8 hypoperfused for 1 month analysis. (D) Representative confocal images showing 1ba1 labelling 3 days and 1 month after the onset of cerebral hypoperfusion. Scale bar = 10 µm. (E) Numbers of microglia were unchanged following 3 days of cerebral hypoperfusion. (F) Following 1 month of cerebral hypoperfusion, a significant increase in microglial number was observed compared to sham controls. n = 13 sham, 12 hypoperfused for 3 day analysis; n = 9 sham, 10 hypoperfused for 1 month analysis.

Despite an absence of gross myelin alterations, in our group we have consistently determined that cerebral hypoperfusion results in a disruption of axon-glial integrity [12], [25], [26] and thus sought to verify whether similar alterations occur in the white matter in this study. Axon-glial integrity was assessed by myelin associated glycoprotein (MAG), a key myelin protein involved in the maintenance of axon-glial integrity (Figure 6A). There was a reduction in the density of MAG staining in the corpus callosum after 3 days (p = 0.008) and 1 month (p = 0.027) of chronic cerebral hypoperfusion (Figures 6B and 6C).

A pronounced microglial response as determined by Iba1 immunoreactivity has also been a robust finding in our model of hypoperfusion (Figure 6D). In support of this after 1 month of hypoperfusion, numbers of microglia were significantly increased compared to sham controls (p = 0.0011) (Figure 6E), whilst numbers of microglia were unchanged after 3 days of cerebral hypoperfusion (p = 0.425) (Figure 6F), consistent with earlier studies using this mouse model [12], [28].

Taken together these data indicate that whilst gross myelin morphology remains intact and increased numbers of oligodendrocytes are observed following hypoperfusion, axon-glial integrity is impaired supporting our previous observations [25].

## Discussion

Previous studies have demonstrated the susceptibility of oligodendrocytes to severe reductions in cerebral blood flow (>70% to that of baseline levels) with profound oligodendrocyte loss occurring early in response to the insult [6], [17]. The present study additionally highlights the vulnerability of these oligodendroglial cells to more modest reductions in blood flow comparable to those observed in the ageing brain.

In the present study we investigated the pools of OPCs and mature oligodendrocytes. We observed two different populations of NG2^+^ cells identified by differences in morphology. One population of NG2^+^ cells showed circular reactivity around the nucleus and few processes, corresponding to ‘early’ stage OPCs, whilst the other population of cells was more intensely stained and more processed, corresponding to ‘late’ stage OPCs. Interestingly only the ‘early’ stage OPCs were affected by hypoperfusion. It is possible that these early OPCs have responded rapidly to hypoperfusion by extending more processes and have thus been classified as late OPCs. Mature oligodendrocytes were also reduced in response to hypoperfusion, highlighting the vulnerability of these glial cells to even modest reductions in cerebral blood flow. Although the mechanisms underlying this early oligodendroglial loss with hypoperfusion remain to be determined, these may involve damage to the oligodendrocytes and OPCs as a result of oxidative stress [31], [32] or inflammation [33], [34], both of which are known inducers of oligodendroglial damage and/or death (for review see [9], [35]). In the present study, consistent with our previous work, we demonstrated a marked microglial response with hypoperfusion. Previously we have also shown alterations in indices of hypoxia [25] in white matter after hypoperfusion. Localised alterations in glutamate levels as a result of compromised blood flow could also contribute to the loss of oligodendrocytes and OPCs via NMDA receptor activation leading to intracellular Ca^2+^-dependent injury to oligodendroglia [36], [37]. However it should be noted that whilst we propose that the loss of CC1+ and NG2+ labelling of cells is an indicator of cell loss, a loss of cellular antigenicity could also account for the reduction in cellular staining.

With sustained hypoperfusion, marked alterations in oligodendroglial pools were observed. The OPC pool was restored and numbers of mature oligodendrocytes were increased when examined after 1 month hypoperfusion. This suggests that there is sufficient capacity with the adult brain to overcome the initial loss of oligodendrocyte pools. Similarly, in models of focal cerebral ischaemia when either blood flow is restored with reperfusion or in the peri-infarct region where there is sufficient collateral flow, increased numbers of OPCs are detectable [16]. In contrast, other studies of mouse cerebral hypoperfusion have shown that oligodendrocyte numbers remain reduced at one month of hypoperfusion [38], [39]. There are notable differences between the model of cerebral hypoperfusion in our group compared to others [28], [38], [39]. Importantly we do not detect demyelination but instead a robust disruption of axon-glial integrity and a pronounced microglial response in white matter [12], [25], [26]. There may also be differences in the time course of progression of oligodendrocyte changes between our studies and others. The level of reduction in cerebral blood flow may be a critical factor which influences the extent of pathology and proliferation/differentiation response. We used laser speckle imaging and demonstrated that in our hands the reduction in CBF was approximately 36% that of baseline at 3 days and then restored to 22% that of shams at 1 month. These are slightly greater than the levels reported previously by Shibata et al. 2004 [28] where the maximal levels of CBF reduction as assessed by laser Doppler flowmetry were 30% although are consistent with more recent studies such as that by Duan et al. [40] who have reported reductions of 37% from that of baseline using Laser Speckle imaging. However, there are a number of other factors that may influence the outcome and differences in pathology in models between different laboratories including the anaesthetic used; the background strain of mice (influences differences in cerebrovasculature) and environment (pathogen status, temperature). Notably however, in these studies that report sustained reductions in oligodendrocyte numbers [38], [39], [41], [42] the pools can be restored by either pharmacological intervention [39], [41], [42] or bone marrow cell treatment [38] indicating that there is restorative capacity of oligodendrocytes in the model. In a previous study we detected a marked increase in genes associated with cell proliferation in white matter in response to hypoperfusion [25] and as a consequence expected to observe marked increases in cell proliferation. However, assessment using the acute proliferation marker PCNA showed that overall levels of proliferation were unchanged with hypoperfusion but revealed that proliferation of Olig2^+^ cells was decreased in 3 day hypoperfused animals compared to controls. It is important to note that whilst proliferation of OPCs was reduced after 3 days, there may be a proliferative response of other cells within the white matter although characterisation of this was beyond the scope of the current study.

To further characterise the early proliferative responses to cerebral hypoperfusion, BrdU incorporation was used to assess the total number of proliferating cells during the first 3 days after the onset of hypoperfusion. This revealed that proliferating cells were detectable in 50% of the hypoperfused cohort compared to 22% of sham operated animals suggesting a modest proliferative response early in response to hypoperfusion. However, this low level of proliferation would be insufficient to account for the restoration of the oligodendroglial pool. It is possible that the BrdU labelling protocol used in this study has not adequately labelled all proliferating cells and thus has underestimated the extent of proliferation. Although it has been reported that BrdU at 200 mg/kg body weight is a saturating dose and does not result in cytotoxicity [43], the dosage used in this study (70 mg/kg body weight/day) is within the standard range of 50–100 mg/kg reported in many studies. However, the use of only two injections per day coupled with the short bioavailability of BrdU may have been insufficient to label all proliferating cells within the injection period. In addition, a comprehensive characterisation of cell proliferation would require continuous administration of BrdU (for example in drinking water) and assessment of BrdU^+^ cells at various time points.

The lack of convincing evidence of proliferation in response to cerebral hypoperfusion raises the question as to whether differentiation of pre-existing OPCs may contribute to the restoration of oligodendrocyte pools. Consistent with the hypothesis that glutamate and ATP may be involved in white matter disruption following cerebral hypoperfusion, there is growing evidence implicating these two neurotransmitters in the regulation of OPC proliferation and differentiation. *In vitro* studies have demonstrated that glutamate inhibits OPC proliferation [21] but promotes OPC differentiation via NMDA receptor activation [44]. Similarly it has been demonstrated that ATP and related derivatives also inhibit proliferation whilst promoting OPC differentiation [20]. Therefore it is possible that the low numbers of proliferating cells in this study may be due to extracellular ATP and glutamate acting to limit OPC proliferation whilst promoting differentiation. In the current study we have demonstrated that a small proportion of newly generated cells had differentiated into mature oligodendrocytes however this is unlikely to exclusively account for the significant increase in oligodendrocyte numbers observed after 1 month. It is possible that differentiation of pre-existing OPCs has also occurred to boost oligodendrocyte numbers in response to cerebral hypoperfusion. Interestingly, this is consistent with a study in a model of cerebral ischaemia which similarly showed repopulation of the oligodendrocyte pool in the absence of significant OPC proliferation [6].

To investigate a potential mechanism involved in differentiation of OPCs in response to hypoperfusion we examined the expression of GPR17, a novel dual uracil nucleotide and cysteinyl-leukotriene G protein-coupled receptor which has been implicated in mediating OPC responses to injury such as ischaemia [22]. It has previously been demonstrated that expression of GPR17 is upregulated in response to ischaemia and it has been suggested that receptor activation may act as a sensor of local damage [22]–[24]. In the current study, we have shown that numbers of GPR17-expressing cells were unchanged but expression of the receptor was decreased during the acute response to cerebral hypoperfusion. There is however conflicting evidence regarding the effects of receptor activation on OPC differentiation. Studies using transgenic mice have shown that receptor over-expression results in decreased myelination whereas knockout leads to increased myelination suggesting that GPR17 is a negative regulator of OPC differentiation [45]. In contrast, it has been shown *in vitro* that receptor agonism results in increased numbers of mature oligodendrocytes, implying that receptor activation promotes differentiation, whilst antagonism increases the proportion of OPCs [24]. Additionally it has also been demonstrated that when cultured OPCs are transferred to medium which promotes differentiation, GPR17 expression is increased [46]. Despite this apparently permissive role of GPR17 in OPC differentiation, it has also been reported that receptor expression may also increase the susceptibility of a cell to ATP-induced death [46]. Thus although GPR17 activation does not appear to mediate OPC responses to cerebral hypoperfusion at the time points examined, its downregulation after 3 days may represent an attempt to limit cell damage and/or death induced by hypoperfusion.

Myelinated axons in the corpus callosum were examined at the ultrastructural level to determine whether alterations to oligodendrocyte numbers had an impact on myelin sheaths. We observed no significant differences in g-ratio, an index of myelin sheath thickness, between sham and 3 day hypoperfused mice despite hypoperfusion inducing around a 15% reduction in mature oligodendrocytes at this time point. Furthermore there were no differences in myelin levels as assessed by fluoromyelin-labelling of myelin in the corpus callosum or MBP levels measured in myelin-enriched extracts. Limitations of these approaches may have precluded detection of myelin alterations after hypoperfusion. In the present study, fluoromyelin staining was conducted in thick sections using a slightly modified protocol to that of previously published work which did detect alterations in myelin in the hypoperfusion model [38], [41]. Thus, differences in fluoromyelin staining methods might explain the different outcomes. Additionally, the present study assessed MBP alterations in myelin-enriched fractions [29] prepared from both white matter and grey matter. Arguably there may have been a dilutional effect of the grey matter on the measurement of MBP levels since this model has been shown to exhibit predominantly a white matter pathology without robust grey matter pathology within 1 month after the surgery [12], [27], [28]. However, this finding (loss of oligodendrocytes and absence of myelin changes), is consistent with a recent study which used diphtheria toxin to specifically ablate oligodendrocyte numbers by approximately 26% and reported that myelin was preserved despite this extensive oligodendrocyte loss [47]. This lack of effect on myelination despite profound alterations in oligodendrocyte pools is also consistent with previous studies by our group which have determined that there are no gross alterations to the protein levels of myelin basic protein as assessed by immunohistochemistry [25]. Instead, in this model, hypoperfusion results in disruption of MAG and breakdown of axon-glial integrity associated with disruption of the paranodal septate junctions [25]. The current study used an antibody against the CC1 protein which labels oligodendrocyte cell bodies but not processes, and as a result cannot reliably distinguish between myelinating and non-myelinating oligodendrocytes. One possible explanation for our findings is that in response to cerebral hypoperfusion, a non-myelinating population of CC1^+^ oligodendrocytes are lost and thus no disruption to myelin has been observed. Another possibility is that neighbouring oligodendrocytes may compensate for oligodendrocyte loss by extending processes and ‘filling in’ non-myelinated areas [48]. We also observed that g-ratio was similarly unchanged following 1 month of hypoperfusion and so it remains to be determined whether increased numbers of oligodendrocytes observed at this time point are surplus to requirements. Overall these findings suggest that even in the presence of cerebral hypoperfusion, the CNS can tolerate significant alterations to mature oligodendrocyte pools without any apparent detriment to myelin thickness or density.

Thus in conclusion this study provides further support that oligodendrocytes are vulnerable to modest blood flow reductions and evidence supporting their regenerative capacity. This turnover of the pool of oligodendrocytes appears to occur in the absence of changes in myelination but it would be interesting to investigate in future studies whether there are any functional consequences of these changes within the white matter.

## Supporting Information

Figure S1 **Reduced cerebral blood flow in hypoperfused animals.** Cerebral blood flow was measured using laser speckle flowmetry prior to surgery (baseline) and at 3 days and 1 month following surgery to assess the extent of hypoperfusion at the times white matter alterations were investigated. (A) Representative images showing speckle images at baseline, 3 days and 1 month for a sham and hypoperfused mouse. Images show the average of 100 frames. Baseline CBF was not significantly different (p<0.05) between groups (1130±36 perfusion units in shams vs 1072±24 perfusion units in hypoperfused group). (B&C) After 3 days and 1 month, CBF was significantly decreased in hypoperfused compared to sham animals to (p<0.001 and p<0.005 respectively). Data are calculated for each mouse as the percentage change relative to baseline. (B) Cerebral blood flow was decreased by approximately 36% to that of shams following 3 days of cerebral hypoperfusion. (C) Following 1 month of hypoperfusion, CBF values had recovered to approximately 22% of sham levels. Data is shown for each mouse, n = 8 sham, 6 hypoperfused.
(TIF)

Figure S2 **NG2 is a specific marker of OPCs.** NG2 and PDGFRα labelling confirmed the specificity of NG2 as an OPC marker in the corpus callosum. (A) NG2 and PDGFRα co-labelled OPCs in the corpus callosum. Scale bar = 10 µm. (B) Confocal images showing representative NG2 and PDGFRβ labelling. No PDGFRβ^+^ labelling of pericytes was observed in the corpus callosum and only occasional PDGFRβ^+^ labelling was observed in the cortex. No NG2^+^/PDGFRβ^+^ cells were observed in either region. Scale bar = 20 µm.
(TIF)

Figure S3 **Low magnification images of NG2, CC1, Olig2 and GPR17 labelling.** (A) Low magnification confocal images showing NG2^+^ labelling of OPCs in the corpus callosum (CC). (B) Confocal images showing CC1^+^ labelling of mature oligodendrocytes in the corpus callosum. (C) Confocal images showing Olig2^+^ labelling of oligodendroglia in the corpus callosum. (D) Confocal images showing GPR17^+^ labelling in the corpus callosum. Scale bars = 50 µm. CTx = Cortex, CC = corpus callosum, CPu = caudate putamen.
(TIF)

Figure S4 **Astrocytes are not labelled with CC1 and numbers are unchanged following 1 month of cerebral hypoperfusion.** It has been reported that a subpopulation of astrocytes can express the CC1 (APC) antigen, therefore CC1 and GFAP double labelling was carried out to determine numbers of CC1^+^ astrocytes. (A) Representative confocal images showing CC1^+^/GFAP^+^ double labelling in the corpus callosum (CC). Scale bar = 50 µm, inset scale bar = 10 µm. Cell counts of numbers of double labelled cells revealed approximately 0.8% of CC1^+^ cells expressed GFAP thus confirming the high specificity of CC1 as a marker of mature oligodendrocytes. (B) Representative confocal images showing GFAP^+^ labelling of astrocytes in the corpus callosum (CC). Scale bar = 50 µm. (C) Numbers of GFAP^+^ astrocytes are unchanged following 1 month of chronic cerebral hypoperfusion. CTx = Cortex, CC = corpus callosum, CPu = caudate putamen.
(TIF)

Figure S5 **GPR17 is expressed by oligodendroglia and a small number of microglia.** (A) Representative confocal images showing GPR17^+^/Olig2^+^ double labelling in the corpus callosum. Arrows indicate double labelled cells. (B) Representative confocal images showing GPR17^+^/Iba1^+^ labelling in the corpus callosum. (C) Cell counting revealed approximately 64.4±3.33% of GPR17^+^ cells express Olig2 in the corpus callosum. Only 8.29±0.75% of GPR17^+^ cells co-expressed Iba1. Scale bars = 50 µm, inset scale bars = 10© µm. CTx = Cortex, CC = corpus callosum, CPu = caudate putamen.
(TIF)

Figure S6 **MBP levels are unchanged following 1 month of cerebral hypoperfusion.** (A) Representative Western blot from myelin enriched extracts showing the different MBP isoforms (25–16 kDa) and GAPDH, the later used as a loading control. (B) Analysis of fluorescent intensity relative to GAPDH showed no significant changes in MBP levels after 1 month of cerebral hypoperfusion. n = 5 sham, n = 8 hypoperfused.
(TIF)
