# Supplementary figures and images for: Restoration of Oligodendrocyte Pools in a Mouse Model of Chronic Cerebral Hypoperfusion
Source: PLoS One. 2014 Feb 3;9(2):e87227. doi: 10.1371/journal.pone.0087227 (PMC3911923; doi:10.1371/journal.pone.0087227)

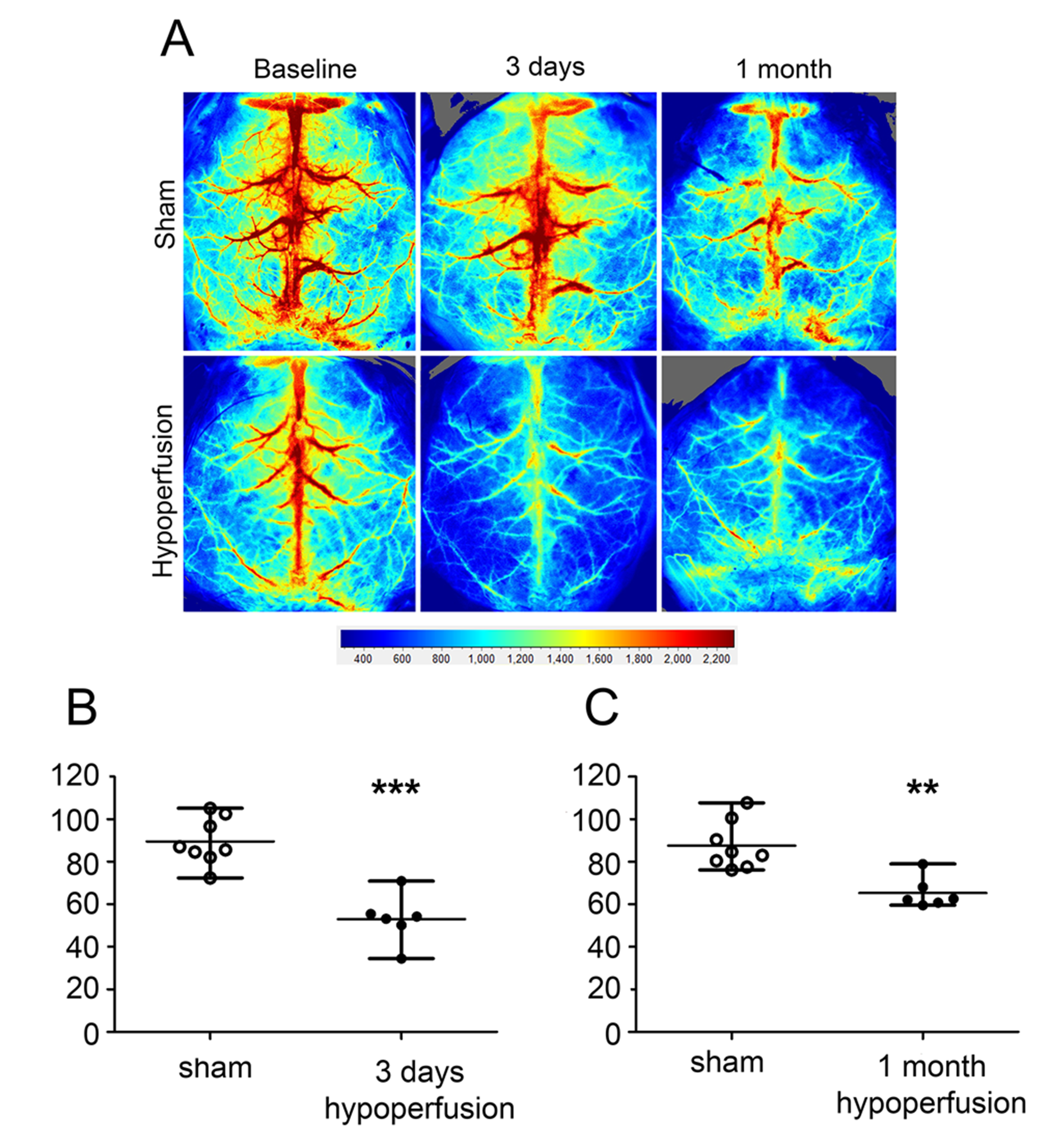

Supplement: Figure S1 — Reduced cerebral blood flow in hypoperfused animals. Cerebral blood flow was measured using laser speckle flowmetry prior to surgery (baseline) and at 3 days and 1 month following surgery to assess the extent of hypoperfusion at the times white matter alterations were investigated. (A) Representative images showing speckle images at baseline, 3 days and 1 month for a sham and hypoperfused mouse. Images show the average of 100 frames. Baseline CBF was not significantly different (p<0.05) between groups (1130±36 perfusion units in shams vs 1072±24 perfusion units in hypoperfused group). (B&C) After 3 days and 1 month, CBF was significantly decreased in hypoperfused compared to sham animals to (p<0.001 and p<0.005 respectively). Data are calculated for each mouse as the percentage change relative to baseline. (B) Cerebral blood flow was decreased by approximately 36% to that of shams following 3 days of cerebral hypoperfusion. (C) Following 1 month of hypoperfusion, CBF values had recovered to approximately 22% of sham levels. Data is shown for each mouse, n = 8 sham, 6 hypoperfused. (TIF) [file pone.0087227.s001.tif]

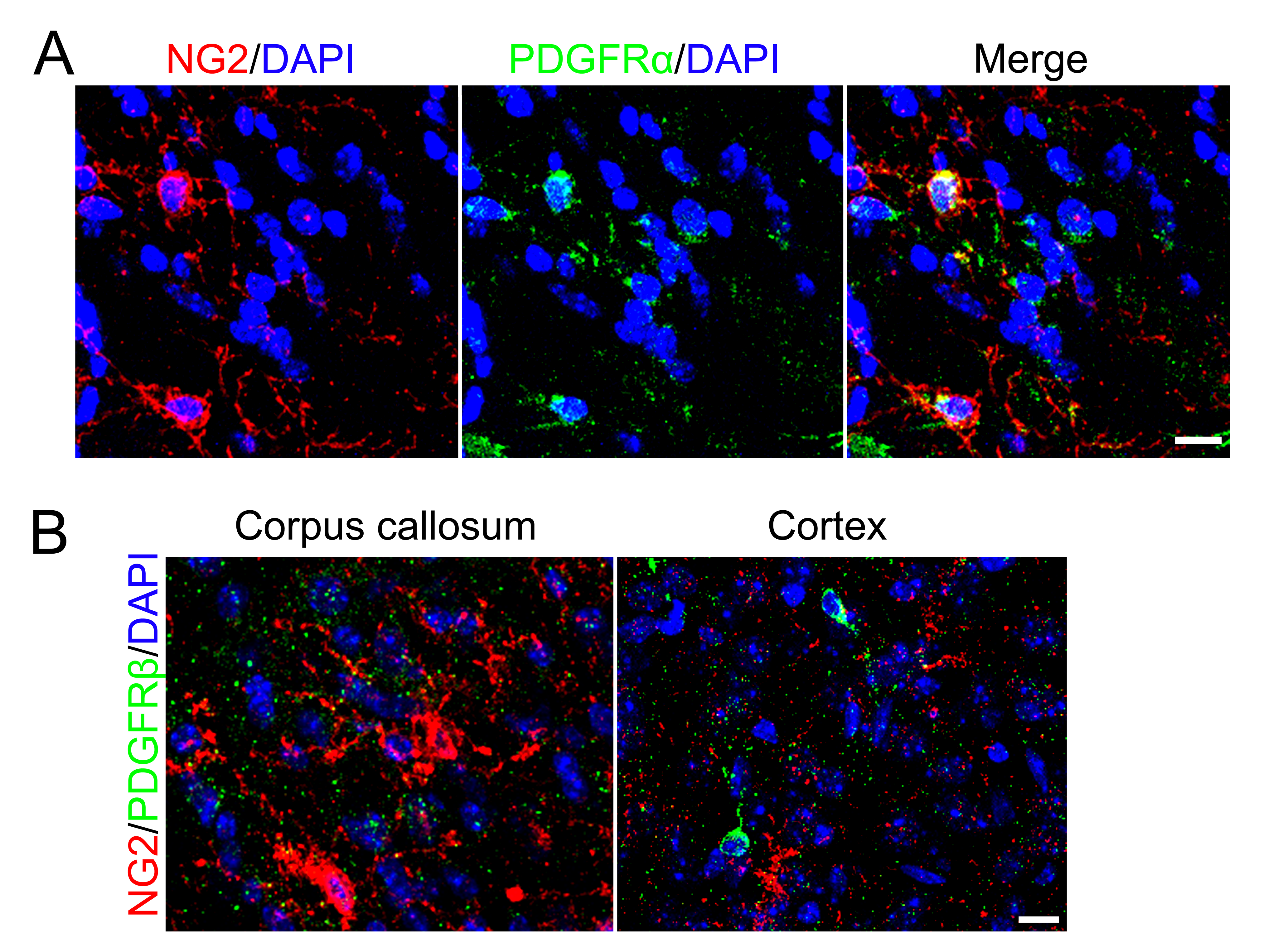

Supplement: Figure S2 — NG2 is a specific marker of OPCs. NG2 and PDGFRα labelling confirmed the specificity of NG2 as an OPC marker in the corpus callosum. (A) NG2 and PDGFRα co-labelled OPCs in the corpus callosum. Scale bar = 10 µm. (B) Confocal images showing representative NG2 and PDGFRβ labelling. No PDGFRβ+ labelling of pericytes was observed in the corpus callosum and only occasional PDGFRβ+ labelling was observed in the cortex. No NG2+/PDGFRβ+ cells were observed in either region. Scale bar = 20 µm. (TIF) [file pone.0087227.s002.tif]

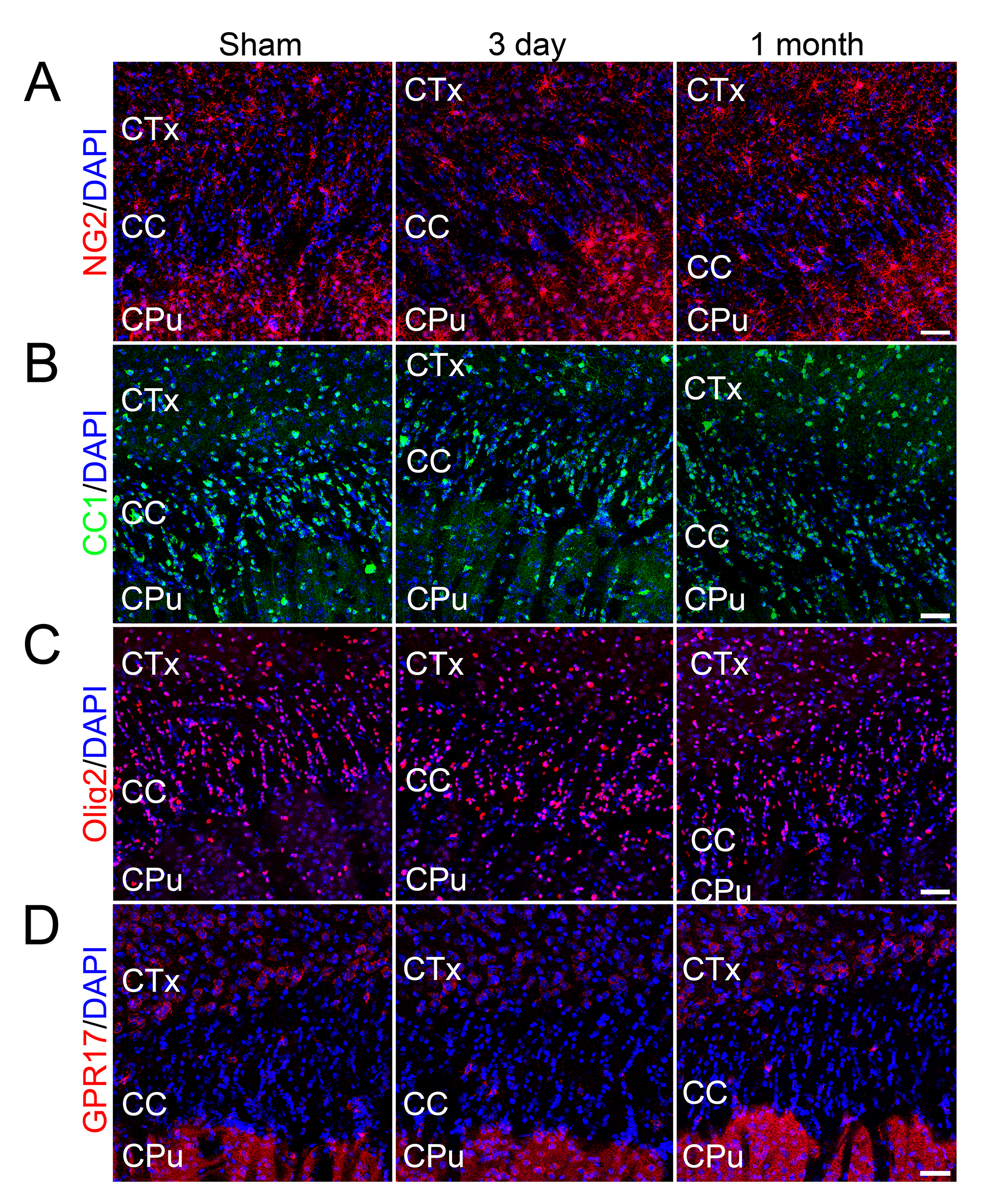

Supplement: Figure S3 — Low magnification images of NG2, CC1, Olig2 and GPR17 labelling. (A) Low magnification confocal images showing NG2+ labelling of OPCs in the corpus callosum (CC). (B) Confocal images showing CC1+ labelling of mature oligodendrocytes in the corpus callosum. (C) Confocal images showing Olig2+ labelling of oligodendroglia in the corpus callosum. (D) Confocal images showing GPR17+ labelling in the corpus callosum. Scale bars = 50 µm. CTx = Cortex, CC = corpus callosum, CPu = caudate putamen. (TIF) [file pone.0087227.s003.tif]

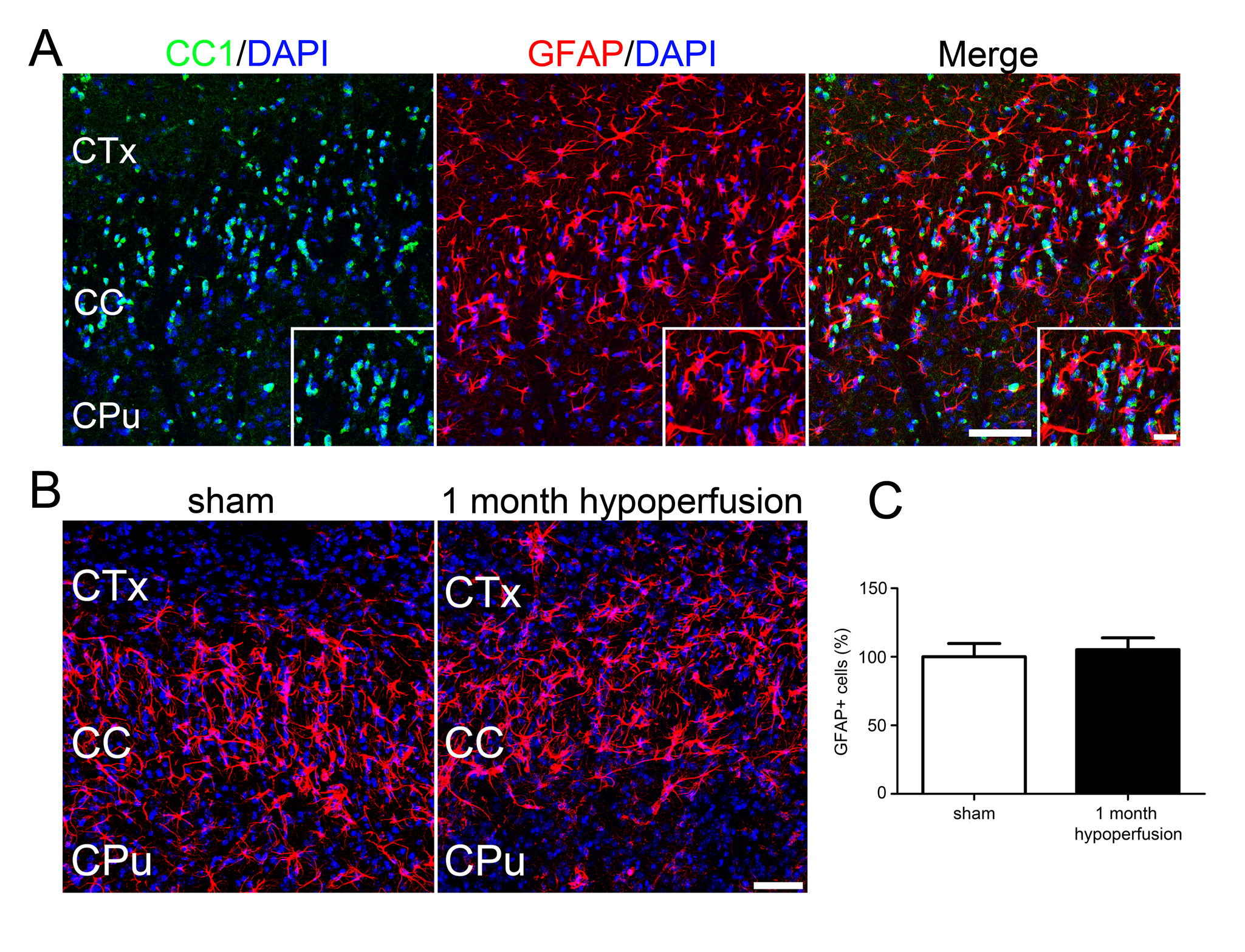

Supplement: Figure S4 — Astrocytes are not labelled with CC1 and numbers are unchanged following 1 month of cerebral hypoperfusion. It has been reported that a subpopulation of astrocytes can express the CC1 (APC) antigen, therefore CC1 and GFAP double labelling was carried out to determine numbers of CC1+ astrocytes. (A) Representative confocal images showing CC1+/GFAP+ double labelling in the corpus callosum (CC). Scale bar = 50 µm, inset scale bar = 10 µm. Cell counts of numbers of double labelled cells revealed approximately 0.8% of CC1+ cells expressed GFAP thus confirming the high specificity of CC1 as a marker of mature oligodendrocytes. (B) Representative confocal images showing GFAP+ labelling of astrocytes in the corpus callosum (CC). Scale bar = 50 µm. (C) Numbers of GFAP+ astrocytes are unchanged following 1 month of chronic cerebral hypoperfusion. CTx = Cortex, CC = corpus callosum, CPu = caudate putamen. (TIF) [file pone.0087227.s004.tif]

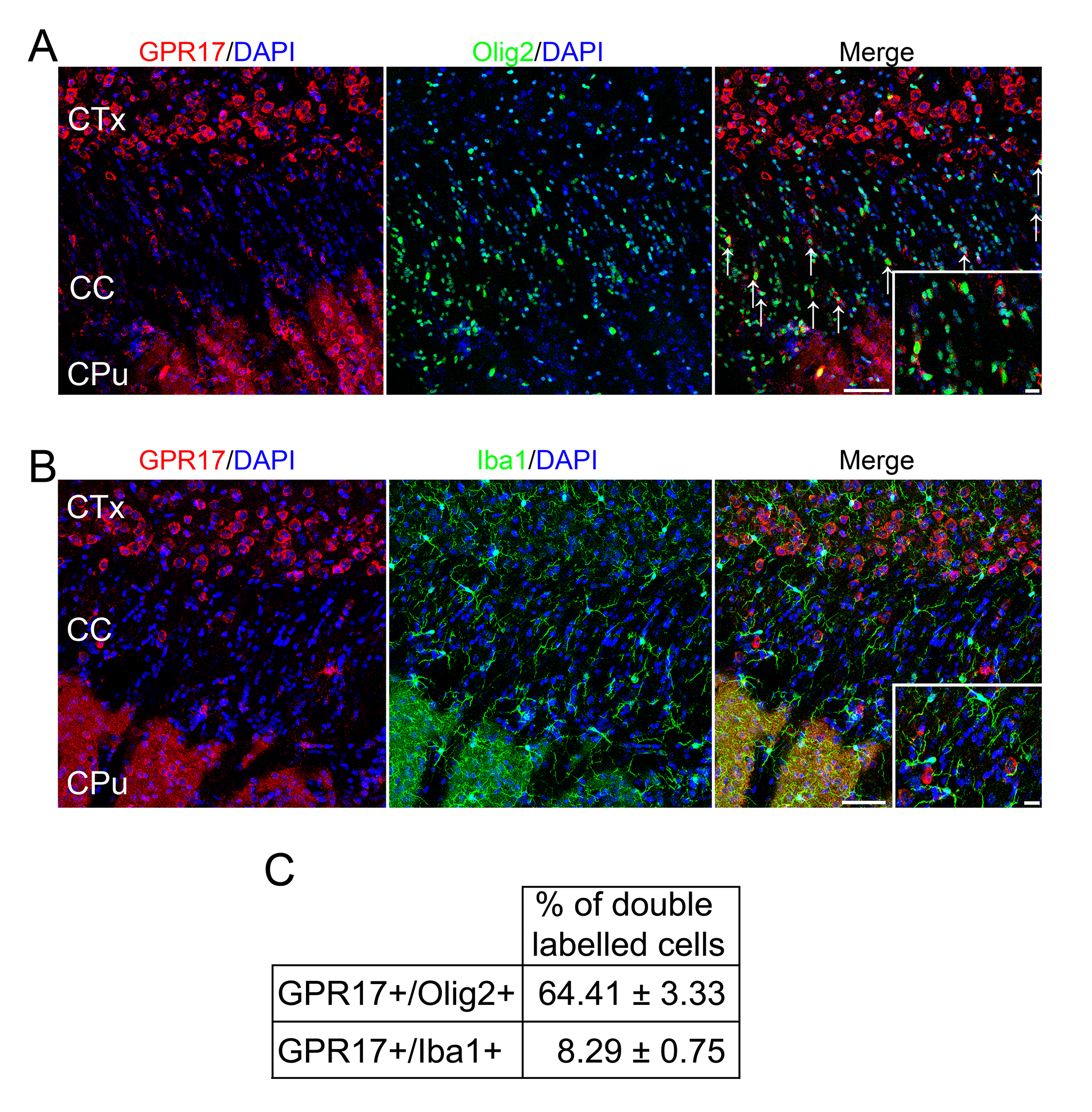

Supplement: Figure S5 — GPR17 is expressed by oligodendroglia and a small number of microglia. (A) Representative confocal images showing GPR17+/Olig2+ double labelling in the corpus callosum. Arrows indicate double labelled cells. (B) Representative confocal images showing GPR17+/Iba1+ labelling in the corpus callosum. (C) Cell counting revealed approximately 64.4±3.33% of GPR17+ cells express Olig2 in the corpus callosum. Only 8.29±0.75% of GPR17+ cells co-expressed Iba1. Scale bars = 50 µm, inset scale bars = 10© µm. CTx = Cortex, CC = corpus callosum, CPu = caudate putamen. (TIF) [file pone.0087227.s005.tif]

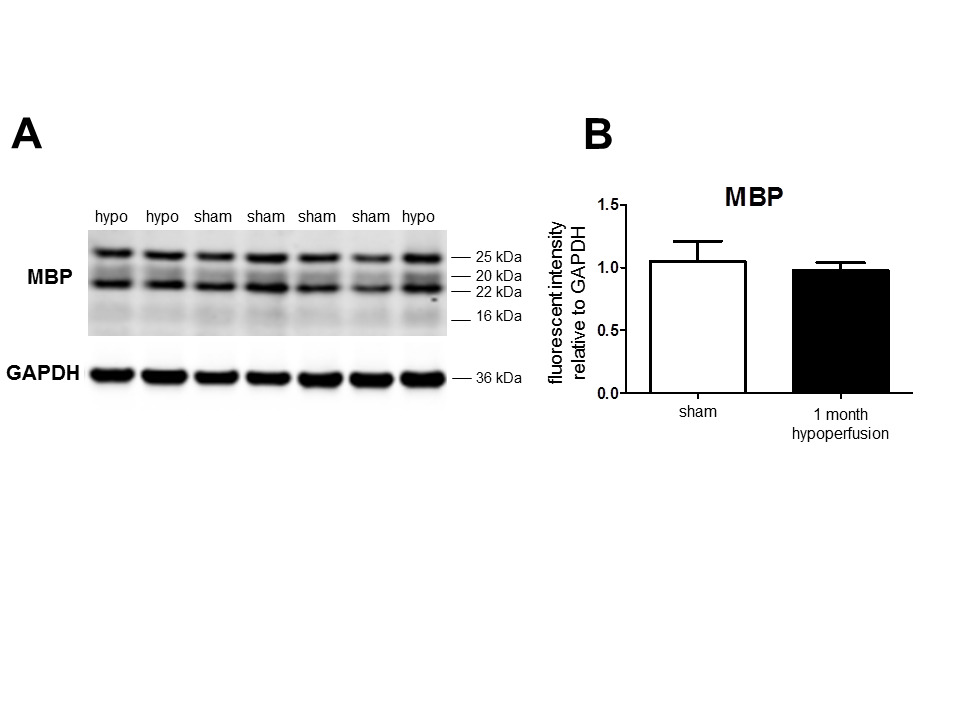

Supplement: Figure S6 — MBP levels are unchanged following 1 month of cerebral hypoperfusion. (A) Representative Western blot from myelin enriched extracts showing the different MBP isoforms (25–16 kDa) and GAPDH, the later used as a loading control. (B) Analysis of fluorescent intensity relative to GAPDH showed no significant changes in MBP levels after 1 month of cerebral hypoperfusion. n = 5 sham, n = 8 hypoperfused. (TIF) [file pone.0087227.s006.tif]
